# *GNAS, PDE4D*, and *PRKAR1A* Mutations and *GNAS* Methylation Changes Are Not a Common Cause of Isolated Early-Onset Severe Obesity Among Finnish Children

**DOI:** 10.3389/fped.2020.00145

**Published:** 2020-04-07

**Authors:** Petra Loid, Minna Pekkinen, Monica Reyes, Taina Mustila, Heli Viljakainen, Harald Jüppner, Outi Mäkitie

**Affiliations:** ^1^Children's Hospital, University of Helsinki and Helsinki University Hospital, Helsinki, Finland; ^2^Folkhälsan Research Center, Genetics Research Program, Helsinki, Finland; ^3^Research Program for Clinical and Molecular Metabolism, Faculty of Medicine, University of Helsinki, Helsinki, Finland; ^4^Endocrine Unit and Pediatric Nephrology Unit, Massachusetts General Hospital and Harvard Medical School, Boston, MA, United States; ^5^Department of Pediatrics, Seinäjoki Central Hospital, Seinäjoki, Finland; ^6^City of Turku, Welfare Division, Preventive Healthcare, Turku, Finland; ^7^The Department of Food and Nutrition, University of Helsinki, Helsinki, Finland; ^8^Department of Molecular Medicine and Surgery, Karolinska Institutet, and Department of Clinical Genetics, Karolinska University Hospital, Stockholm, Sweden

**Keywords:** *GNAS*, G protein-cAMP-signaling, childhood-onset obesity, pseudohypoparathyroidism, acrodysostosis

## Abstract

**Context:** Pseudohypoparathyroidism type Ia (PHP1A) is caused by inactivating mutations involving *GNAS* exons 1–13, encoding the alpha-subunit of the stimulatory G protein (Gsα). Particularly PHP1A, but also other disorders involving the Gsα-cAMP-signaling pathway, have been associated with early-onset obesity. Thus, patients with mutations in the genes encoding PDE4D and PRKAR1A can also be obese. Furthermore, epigenetic *GNAS* changes, as in pseudohypoparathyroidism type Ib (PHP1B), can lead to excessive weight.

**Objective:** Search for genetic variants in *GNAS, PDE4D*, and *PRKAR1A* and for methylation alterations at the *GNAS* locus in Finnish subjects with isolated severe obesity before age 10 years.

**Methods:** Next generation sequencing to identify pathogenic variants in the coding exons of *GNAS, PDE4D*, and *PRKAR1A*; Multiplex Ligation-dependent Probe Amplification (MLPA) and methylation-sensitive MLPA (MS-MLPA) to search for deletions in *GNAS* and *STX16*, and for epigenetic changes at the four differentially methylated regions (DMR) within *GNAS*.

**Results:** Among the 88 subjects (median age 13.8 years, median body mass index Z-score +3.9), we identified one rare heterozygous missense variant of uncertain significance in the XL exon of *GNAS* in a single patient. We did not identify clearly pathogenic variants in *PDE4D* and *PRKAR1A*, and no *GNAS* methylation changes were detected by MS-MLPA.

**Conclusions:** Our results suggest that coding *GNAS* mutations or methylation changes at the *GNAS* DMRs, or coding mutations in *PDE4D* and *PRKAR1A* are not common causes of isolated childhood obesity in Finland.

## Introduction

Pseudohypoparathyroidism (PHP) and related disorders have been associated with early-onset obesity (1, 2). These diseases, which can present with highly variable clinical findings (3), are caused by a defect in the stimulatory G protein-cAMP-signaling pathway.

*GNAS* is a complex imprinted locus on chromosome 20q13.3. Exons 1–13 encode the alpha-subunit of the stimulatory G protein (Gsα), a signaling protein mediating the functions of several hormones that require the second messenger cAMP down-stream of their cognate receptors (3). *GNAS* also encodes several other transcripts with incompletely defined biological actions, including the mRNAs encoding the neuroendocrine secretory protein 55 (NESP55) and the extra-large form of Gsα (XLαs), as well as the A/B and the antisense (AS) transcript.

The Gsα transcript is biallelically expressed in most but not all tissues; the paternal expression is reduced in several tissues, e.g., proximal renal tubules, thyroid, gonads, brown adipose tissue, hypothalamus, and pituitary gland (3). Clinical and laboratory manifestations of *GNAS* mutations are therefore determined by the parental origin of the mutated allele. Mutations involving exons 1–13 of the maternal *GNAS* allele underlie pseudohypoparathyroidism type 1A (PHP1A) in which patients develop resistance to PTH in the proximal renal tubules leading to elevated plasma PTH levels, hypocalcemia and hyperphosphatemia, and often resistance to multiple other hormones. Patients with PHP1A show clinical features of Albright's hereditary osteodystrophy (AHO), including short stature, brachydactyly, early-onset obesity, neurodevelopmental defects and subcutaneous ossifications. In contrast, inactivating mutations located on the paternal *GNAS* allele cause pseudopseudohypoparathyroidism (PPHP) characterized in most patients by several AHO features, but no hormonal resistance, no obesity, and no intellectual challenges. Methylation defects at *GNAS* exon A/B alone, or at several *GNAS* DMRs, are observed in pseudohypoparathyroidism type 1B (PHP1B), characterized by resistance to PTH and frequently TSH, but infrequently by AHO features (3).

Other genes involved in the Gsα-cAMP-signaling pathway are *PRKAR1A* and *PDE4D*. *PRKAR1A* encodes the regulatory subunit of cAMP-dependent protein kinase A (PKA) and *PDE4D* encodes the phosphodiesterase 4D. Heterozygous mutations in *PDE4D* and *PRKAR1A* lead to acrodysostosis because cAMP is either degraded too rapidly or unable to dissociate the regulatory from the catalytic subunit of PKA. The disorders caused by mutations in the latter genes present with clinical manifestations overlapping partly those encountered in PHP (4).

Early-onset obesity can be the first and only evidence for pseudohypoparathyroidism (2, 5). Several mechanisms can be responsible for the excessive weight gain. In patients with PHP1A, reduced energy expenditure caused by the decreased Gsα-mediated signaling in brown or beige fat cells, as well as impaired signaling at the melanocortin 4 receptor leading to hyperphagia and thus increased caloric intake have been proposed as important mechanisms leading to obesity (6–8). Furthermore, the resistance to TSH and growth hormone-releasing-hormone may play a role in the development of obesity (2). Early-onset and childhood obesity has also been observed in PHP1B (1, 9). Based on these observations, it has been proposed that genetic and epigenetic defects at the *GNAS* locus should be considered in patients with unexplained childhood obesity (2). In fact, mutations in the *GNAS* exons encoding Gsα were recently identified in a large British cohort of obese patients (10). This prompted us to look for genetic variants in *GNAS, PDE4D*, and *PRKAR1A*, and for methylation alterations of *GNAS* locus in a cohort of Finnish patients who had developed severe obesity already in early childhood.

## Materials and Methods

This study included 88 subjects recruited through pediatric endocrine clinics at Children's Hospital at Helsinki University Hospital and Seinäjoki Central Hospital in Finland. The study was approved by the Research Ethics Committees of the Hospital District of Helsinki and Uusimaa and the Pirkanmaa Hospital District. Informed written consent was obtained from all participants or their guardians (subjects aged <18 years).

The inclusion criterion was severe obesity as defined according to the Finnish growth standards (11) as height-adjusted weight >60% before 10 years of age. The patients had been followed up by a pediatrician and patients who were diagnosed with a known underlying endocrine or genetic disorder were excluded from the study. Clinical and growth data were collected from hospital records. Height, weight and waist circumference were measured during a study visit. Sex-and age-specific BMI Z-scores were derived based on the World Health Organization reference values (www.who.int/childgrowth/standards).

Genomic DNA was isolated from peripheral blood samples according to standard procedures. The probes for targeted exome sequencing were designed using SeqCap EZ Choice Library and NimbleDesign (Roche NimbleGen, United States). DNA capture and Next Generation Sequencing were performed at Oxford Genomics Centre. The reads were aligned to the reference genome hg19. The software Platypus version 0.8.1 was used for variant calling and Ensembl Variant Effector Predictor (VEP) was used for functional annotation of the variants. Filtering of variants was performed using VarAFT 2.13 (http://varaft.eu). Exonic and splice site variants were considered. We compared the allele frequencies of the identified variants to those obtained from the Exome Aggregation Consortium (http://exac.broadinstitute.org), the Genome Aggregation Database (http://gnomad.broadinstitute.org), 1,000 Genomes Project (http://www.internationalgenome.org) and the Sequencing Initiative Suomi project (SISu) (http://sisuproject.fi). The potential pathogenicity of the sequence variants was evaluated using different variant prediction databases (Polyphen-2, SIFT, Combined Annotation Dependent Depletion (CADD) and MutationTaster2). Sanger sequencing was performed to confirm findings. Primers were designed using Primer3 software and PCR was performed using DreamTaq^TM^ DNA Polymerase (Thermo Fisher Scientific) according to standard protocol and chromatograms were analyzed with Sequencer v5.0 software. Primer sequences are available upon request.

Multiplex Ligation-dependent Probe Amplification (MLPA) to search for *GNAS* or *STX16* deletions and analysis of the *GNAS* methylation status, as assessed by methylation specific multiplex ligation-dependent probe amplification (MS-MLPA), was performed using kit ME031 GNAS (MRC-Holland, Amsterdam, The Netherland) following the manufacturer's instructions (https://www.mlpa.com/), as described (12). PCR products were examined using the ABI3730xl Genetic Analyzer at the DNA Core Facility of the Massachusetts General Hospital and Peak Scanner 2.0 software (http://peak-scanner-software.software.informer.com/2.0/). The results obtained for two to five probes at each of the different DMRs were averaged for each patient and mean± standard deviation (SD) were subsequently calculated for each DMR for the entire cohort.

## Results

### Baseline Characteristics

The median age for the 88 study subjects (54.5% males) was 13.8 years [interquartile range (IQR) 11.1–17.0 years]. The median BMI Z-score at the time of the study visit was +3.9 (IQR +3.4 to +4.9). The study subjects fulfilled our inclusion criteria of severe obesity (height-adjusted weight >60%) at the median age of 5.3 years (IQR 4.1–7.0 years). They had reached height-adjusted weight >40% (corresponding to obesity according to Finnish growth standards) at the median age of 4.0 years (IQR 2.0–5.0 years) (Table 1). Of the 68 school-aged subjects for whom information on learning difficulties was available, 10 (15%) had learning difficulties and required special education.

**Table 1:** Characteristics of study subjects, *n* = 88.

Male, *n* (%)	48 (54.5)
Age (yrs)	13.8 (11.1-17.0)
BMI Z-score	3.9 (3.4-4.9)
Age when weight-adjusted height 60% (yrs)	5.3 (4.1-7.0)
Age when weight-adjusted height 40% (yrs)	4.0 (2.0-5.0)

*Data presented as median (interquartile range)*.

### Targeted Exome Sequencing

The variants detected in *GNAS, PDE4D*, and *PRKAR1A* are presented in Table 2. We identified one rare heterozygous missense *GNAS* variant in exon XL (c.897C>A, p.S299R) in a female with severe early-onset obesity. This variant is found with allele frequency 0.00036 in the Finnish population in gnomAD. Polyphen-2 predicted the variant as possibly damaging, SIFT as tolerated, MutationTaster2 as disease-causing; the CADD score was high 23. The patient presented with obesity already at 3.5 years of age with weight 24.5 kg and height 105.5 cm (BMI Z-score +3.8). Presently at the age of 21 years, she has severe obesity: weigh 121 kg, height 170.5 cm (BMI 42) and waist circumference 120 cm. The patient did not present with any hormone resistance or clinical characteristics of AHO apart from obesity. Both parents were obese, but their DNA samples were unavailable for genotyping.

**Table 2:** Non-synonymous sequence variants found in *GNAS, PRKAR1A*, and *PDE4D* genes (reference genome hg19).

**Gene**	**Patients (*n*)**	**Variant**	**Transcript**	**rs number**	**MAF obesity cohort**	**MAF gnomAD**	**MAF gnomAD Finnish population**	**CADD**	**SIFT**	**Polyphen-2**	**MutationTaster**
GNAS	1	20:57429217 C>A	NM_080425 (XL) c.897C>A, p.S299R	rs200409817	0.0057	0.0001	0.00036	23	Tolerate	Possibly damaging	Disease causing
GNAS	1	20:57429447 C>T	NM_080425 (XL) c.1127C>T, p.P376L	rs61749697	0.0057	0.02	0.01	22	Tolerate	Benign	Disease causing
GNAS	1	20:57415876 C>A	NM_016592 (NESP55) c.715C>A, p.P239T	rs79527543	0.0057	0.004	0.00	22	Deleterious	Possibly damaging	Polymorphism
GNAS	1	20:57428820 A>G	NM_080425 (XL) c.500A>G, p.D167G	rs61749695	0.0057	0.002	0.00	22	Deleterious	Benign	Disease causing
GNAS	5	20:57429696 C>G	NM_080425 (XL) c.1376C>G, p.P459R	rs148033592	0.0284	0.011	0.003	19	Deleterious	Benign	Polymorphism
GNAS	5	20:57429627 C>A	NM_080425 (XL) c.1307C>A, p.A436D	rs61749698	0.0284	0.06	0.005	0.02	Tolerate	Benign	Polymorphism
PDE4D	1	5:59064277 C>T	NM_001197218, c.59G>A, p.C20Y	rs201360779	0.0057	0.001	0.011	17	Deleterious	Possibly damaging	Disease causing
PRKAR1A	38	17:66547249 G>A	NM_001276290, c.998G>A, p.S333N	rs9789047	0.2136	0.17	0.24	0.02	Not applicable	Not applicable	Polymorphism

*MAF, minor allele frequency*.

Furthermore, five heterozygous variants were identified that change single amino acids in GNAS exon XL and NESP, respectively. However, these variants have allele frequencies in gnomAD comparable to the allele frequencies observed in our cohort. No variants were identified in the *GNAS* exons encoding Gsα. In *PDE4D* or *PRKAR1A*, we did not detect any variants that have previously been shown to be disease-causing.

### Multiplex Ligation-Dependent Probe Amplification

Analysis of *GNAS* methylation by MS-MPLA did not identify any epigenetic alterations at the *GNAS* locus (Supplemental Table 1); the DMRs showed no evidence for abnormal methylation at NESP (53.4 ± 2.5%), AS (53.9 ± 3.1%), XL (53.5 ± 2.9%), and A/B (52.4 ± 3.6%).

## Discussion

Pseudohypoparathyroidism and acrodysostosis are rare, heterogeneous disorders caused by different genetic/epigenetic defects (3). Differential diagnosis between these disorders can be challenging because of their overlapping clinical and/or laboratory phenotypes. The clinical features can be difficult to identify by physical examination and some patients show only minor clinical abnormalities that furthermore can vary with age (13). Furthermore, PTH-resistance in PHP1B may remain undetected until symptomatic hypocalcemia develops (2). Establishing the correct diagnosis can thus be challenging but would be of significant importance because of the multiple endocrine disturbances related to these disorders.

Previous studies have found that obesity is a common clinical finding in children with pseudohypoparathyroidism type 1A (2, 5). In a study investigating early features of PHP1A, the major clinical sign was obesity, present in 70% of children >2 years of age (5). Furthermore, it has been suggested that *GNAS* defects in patients with early-onset obesity may be underestimated, since Hendricks et al. (10) found several novel variants in *GNAS* in unselected patients with severe obesity, yet no obvious endocrine defects or short stature. However, many of the patients in the study by Hendricks et al. with *GNAS* variants had developmental delay (10).

We searched for pathogenic variants in *GNAS, PDE4D*, and *PRKAR1A*, and for *GNAS* methylation changes in 88 Finnish subjects with isolated severe early-onset obesity. We identified only one rare heterozygous missense variant in *GNAS* exon XL in a patient with isolated severe obesity. Unfortunately, parental DNA samples were not available. This variant does not affect the transcript encoding Gsα. The patient did not present with any skeletal, endocrine or developmental defect. The clinical significance of this missense variant thus remains uncertain. We furthermore identified four heterozygous non-synonymous amino acid changes in *GNAS* exon XL and one change in exon NESP, as well as single variants in *PRKAR1A* and in *PDE4D*. All these amino acid changes were present in gnomAD with comparable or higher allele frequencies and were not predicted to be disease-causing variants.

We did not identify any pathogenic defects in the investigated genes. Although we had carefully selected our patients presenting severe obesity with early onset, the relatively small cohort size of only 88 patients can be regarded as a limitation. Furthermore, patients with PHP and related disorders may have been identified earlier by pediatricians and could therefore not be included in our study.

Another limitation of our study was the lack of a control group of normal-weight subjects. However, we compared the allele frequencies of the identified variants with the Finnish population in gnomad and SISu project, which include genotype data of more than 10,000 individuals from the Finnish population.

In conclusion, our results suggest that mutations in *GNAS, PDE4D*, and *PRKAR1A*, and methylation changes in *GNAS* locus do not play a significant role in etiology of childhood-onset obesity in our Finnish cohort of patients, who lacked biochemical features of pseudohypoparathyroidism and related disorders.

## Data Availability Statement

Data cannot be shared publicly because the data consists of sensitive patient data. More specifically the data consists of individual clinical data and individual genotypes for young children. Data are available from the Helsinki University Hospital's Institutional Data Access/Ethics Committee for researchers who meet the criteria for access to confidential data. Data availability contact: Outi Mäkitie MD, PhD.

## Ethics Statement

The study was approved by the Research Ethics Committees of the Hospital District of Helsinki and Uusimaa and the Pirkanmaa Hospital District. Written informed consent to participate in this study was provided by the participants' legal guardian/next of kin.

## Author Contributions

Study design: PL, MP, TM, HV, HJ, and OM. Data collection: PL, TM, and HV. Data analysis and data interpretation: PL, MP, MR, HJ, and OM. Drafting of manuscript: PL, MP, HJ, and OM. All authors reviewed manuscript content and approved the final version.

### Conflict of Interest

The authors declare that the research was conducted in the absence of any commercial or financial relationships that could be construed as a potential conflict of interest.
