# Supplementary material for: GNAS, PDE4D, and PRKAR1A Mutations and GNAS Methylation Changes Are Not a Common Cause of Isolated Early-Onset Severe Obesity Among Finnish Children
Source: Front Pediatr. 2020 Apr 7;8:145. doi: 10.3389/fped.2020.00145 (PMC7155765; doi:10.3389/fped.2020.00145)
Supplement: Supplementary file 1 [file Table_1.DOCX]

| **Supplemental Table 1. Results of GNAS methylation analysis by MS-MLPA** | | | | | | | | | | |  |  |  |  |  |  |  |  |
| --- | --- | --- | --- | --- | --- | --- | --- | --- | --- | --- | --- | --- | --- | --- | --- | --- | --- | --- |
| **Patient** | **NESP55**  **Exon1**  **(197nt)** | **NESP55**  **Exon1**  **(264nt)** | **NESP55**  **Exon1**  **(411nt)** | **mean** | **NESPAS**  **Exon1**  **(166nt)** | **NESPAS**  **Exon1**  **(321nt)** | **NESPAS**  **Exon1**  **(250nt)** | **mean** | **GNAS**  **XL**  **Exon1**  **(300nt)** | **GNAS**  **XL**  **Exon1**  **(160nt)** | | **GNAS**  **XL**  **Exon1**  **(462nt)** | **GNAS**  **XL**  **Exon1**  **(315nt)** | **GNAS**  **XL**  **Intron1**  **(468nt)** | **mean** | **GNAS**  **Exon**  **A/B**  **(238nt)** | **GNAS**  **Exon**  **A/B**  **(401nt)** | **mean** |
| 1 | 49,62 | 54,61 | 49,33 | 51,2 | 53,87 | 54,36 | 54,08 | 54,1 | 58,73 | 55,18 | | 62,53 | 47,94 | 56,93 | 56,3 | 56,70 | 61,10 | 58,9 |
| 2 | 58,16 | 56,20 | 58,38 | 57,6 | 48,73 | 47,17 | 50,42 | 48,8 | 47,38 | 44,92 | | 49,09 | 45,23 | 50,23 | 47,4 | 46,13 | 47,22 | 46,7 |
| 3 | 49,34 | 54,25 | 48,30 | 50,6 | 48,61 | 47,92 | 52,22 | 49,6 | 52,95 | 51,44 | | 51,34 | 49,41 | 53,17 | 51,7 | 49,84 | 48,78 | 49,3 |
| 4 | 51,65 | 48,28 | 51,22 | 50,4 | 53,14 | 48,13 | 52,68 | 51,3 | 53,30 | 54,97 | | 55,81 | 55,72 | 60,10 | 56,0 | 58,63 | 58,05 | 58,3 |
| 5 | 52,04 | 52,80 | 55,79 | 53,5 | 50,19 | 49,42 | 56,18 | 51,9 | 58,04 | 55,53 | | 55,51 | 49,03 | 55,70 | 54,8 | 54,85 | 56,41 | 55,6 |
| 6 | 53,29 | 57,81 | 47,28 | 52,8 | 55,16 | 50,52 | 52,94 | 52,9 | 57,44 | 54,18 | | 50,61 | 46,45 | 49,85 | 51,7 | 48,32 | 48,04 | 48,2 |
| 7 | 57,46 | 54,97 | 51,44 | 54,6 | 52,18 | 52,11 | 65,31 | 56,5 | 52,16 | 58,47 | | 53,05 | 49,75 | 51,83 | 53,1 | 52,24 | 47,62 | 49,9 |
| 8 | 54,55 | 56,13 | 53,22 | 54,6 | 48,15 | 55,17 | 56,77 | 53,4 | 61,24 | 49,68 | | 61,88 | 52,39 | 54,09 | 55,9 | 63,49 | 57,26 | 60,4 |
| 9 | 51,02 | 49,46 | 47,20 | 49,2 | 53,40 | 53,79 | 66,24 | 57,8 | 56,73 | 51,89 | | 51,80 | 46,89 | 58,03 | 53,1 | 58,21 | 59,43 | 58,8 |
| 10 | 52,48 | 54,42 | 60,54 | 55,8 | 58,55 | 58,44 | 57,54 | 58,2 | 59,21 | 56,15 | | 52,17 | 51,30 | 55,19 | 54,8 | 55,22 | 55,33 | 55,3 |
| 11 | 54,44 | 54,79 | 53,36 | 54,2 | 51,56 | 57,44 | 55,44 | 54,8 | 53,69 | 49,81 | | 53,82 | 47,34 | 53,20 | 51,6 | 51,79 | 51,67 | 51,7 |
| 12 | 55,26 | 56,09 | 53,86 | 55,1 | 55,69 | 51,87 | 60,26 | 55,9 | 58,21 | 55,24 | | 56,68 | 50,57 | 57,42 | 55,6 | 51,50 | 49,08 | 50,3 |
| 13 | 55,09 | 52,30 | 52,99 | 53,5 | 50,51 | 50,77 | 55,01 | 52,1 | 56,95 | 60,52 | | 53,86 | 51,68 | 51,72 | 54,9 | 56,42 | 50,40 | 53,4 |
| 14 | 51,27 | 52,42 | 52,57 | 52,1 | 49,11 | 49,19 | 51,40 | 49,9 | 54,03 | 53,53 | | 51,52 | 47,92 | 48,56 | 51,1 | 49,73 | 48,97 | 49,4 |
| 15 | 52,53 | 54,06 | 51,01 | 52,5 | 52,57 | 47,36 | 51,30 | 50,4 | 56,95 | 53,13 | | 52,48 | 54,20 | 52,68 | 53,9 | 53,58 | 50,54 | 52,1 |
| 16 | 50,85 | 50,64 | 53,91 | 51,8 | 50,15 | 49,06 | 56,70 | 52,0 | 54,39 | 56,14 | | 51,19 | 48,95 | 54,17 | 53,0 | 63,06 | 49,84 | 56,4 |
| 17 | 49,56 | 52,93 | 51,51 | 51,3 | 52,66 | 52,55 | 57,48 | 54,2 | 57,25 | 57,37 | | 55,27 | 51,62 | 52,58 | 54,8 | 54,18 | 52,95 | 53,6 |
| 18 | 51,04 | 51,23 | 53,87 | 52,0 | 51,80 | 58,84 | 54,02 | 54,9 | 59,67 | 54,93 | | 56,88 | 52,15 | 49,24 | 54,6 | 53,08 | 58,54 | 55,8 |
| 19 | 46,78 | 56,74 | 55,41 | 53,0 | 48,75 | 56,05 | 55,80 | 53,5 | 55,11 | 50,12 | | 57,66 | 53,39 | 55,45 | 54,3 | 52,60 | 53,64 | 53,1 |
| 20 | 48,68 | 46,25 | 52,20 | 49,0 | 48,74 | 53,32 | 50,25 | 50,8 | 53,69 | 48,31 | | 53,60 | 48,03 | 56,14 | 52,0 | 50,20 | 54,73 | 52,5 |
| 21 | 51,75 | 50,81 | 51,91 | 51,5 | 47,20 | 46,12 | 54,50 | 49,3 | 51,10 | 51,15 | | 53,61 | 50,54 | 54,65 | 52,2 | 52,44 | 51,75 | 52,1 |
| 22 | 57,58 | 50,52 | 49,47 | 52,5 | 57,72 | 52,03 | 50,99 | 53,6 | 54,07 | 48,53 | | 46,12 | 50,90 | 52,34 | 50,4 | 53,39 | 58,15 | 55,8 |
| 23 | 58,32 | 60,34 | 52,00 | 56,9 | 48,96 | 49,58 | 54,70 | 51,1 | 56,39 | 53,62 | | 53,98 | 47,91 | 54,17 | 53,2 | 49,69 | 53,31 | 51,5 |
| 24 | 56,59 | 55,16 | 49,02 | 53,6 | 52,23 | 48,82 | 48,52 | 49,9 | 52,47 | 53,85 | | 52,07 | 51,53 | 56,62 | 53,3 | 49,78 | 54,26 | 52,0 |
| 25 | 59,44 | 52,08 | 48,25 | 53,3 | 55,51 | 47,56 | 57,42 | 53,5 | 50,36 | 57,95 | | 55,11 | 49,16 | 57,02 | 53,9 | 52,70 | 53,78 | 53,2 |
| 26 | 49,45 | 51,67 | 54,30 | 51,8 | 51,85 | 52,35 | 56,43 | 53,5 | 54,89 | 54,41 | | 52,23 | 43,99 | 57,75 | 52,7 | 47,08 | 50,56 | 48,8 |
| 27 | 57,67 | 50,81 | 49,40 | 52,6 | 58,86 | 48,94 | 56,77 | 54,9 | 56,25 | 50,49 | | 57,63 | 47,51 | 53,73 | 53,1 | 55,65 | 53,85 | 54,7 |
| 28 | 47,44 | 57,91 | 48,49 | 51,3 | 50,82 | 52,41 | 54,11 | 52,4 | 59,71 | 50,55 | | 50,91 | 50,77 | 51,27 | 52,6 | 47,48 | 46,27 | 46,9 |
| 29 | 54,56 | 54,80 | 52,09 | 53,8 | 56,25 | 52,29 | 59,01 | 55,9 | 51,86 | 52,72 | | 54,61 | 51,98 | 55,65 | 53,4 | 55,53 | 53,17 | 54,3 |
| 30 | 57,45 | 55,08 | 46,85 | 53,1 | 56,17 | 51,05 | 51,72 | 53,0 | 55,47 | 64,66 | | 50,75 | 45,20 | 50,52 | 53,3 | 53,68 | 46,86 | 50,3 |
| 31 | 47,68 | 49,02 | 52,40 | 49,7 | 49,59 | 46,78 | 48,54 | 48,3 | 51,70 | 50,27 | | 47,42 | 43,12 | 50,71 | 48,6 | 49,49 | 42,03 | 45,8 |
| 32 | 60,79 | 54,79 | 53,28 | 56,3 | 55,33 | 56,46 | 50,89 | 54,2 | 54,11 | 53,60 | | 53,92 | 48,66 | 62,64 | 54,6 | 48,97 | 49,46 | 49,2 |
| 33 | 54,65 | 51,73 | 50,71 | 52,4 | 55,47 | 50,72 | 56,45 | 54,2 | 53,38 | 50,19 | | 51,76 | 40,29 | 51,01 | 49,3 | 46,19 | 44,46 | 45,3 |
| 34 | 61,57 | 54,38 | 57,09 | 57,7 | 59,11 | 57,75 | 57,16 | 58,0 | 62,02 | 57,60 | | 58,19 | 50,08 | 62,53 | 58,1 | 56,87 | 52,33 | 54,6 |
| 35 | 60,56 | 52,98 | 50,25 | 54,6 | 59,16 | 56,39 | 58,32 | 58,0 | 61,21 | 51,84 | | 55,63 | 45,36 | 54,10 | 53,6 | 59,50 | 49,24 | 54,4 |
| 36 | 52,25 | 54,16 | 47,89 | 51,4 | 57,04 | 53,50 | 51,85 | 54,1 | 55,13 | 55,99 | | 52,55 | 49,18 | 52,49 | 53,1 | 60,40 | 49,08 | 54,7 |
| 37 | 50,45 | 49,86 | 43,91 | 48,1 | 61,46 | 49,21 | 53,46 | 54,7 | 56,93 | 52,30 | | 47,83 | 43,29 | 46,33 | 49,3 | 54,40 | 41,96 | 48,2 |
| 38 | 60,51 | 49,40 | 52,23 | 54,0 | 56,74 | 50,54 | 51,52 | 52,9 | 56,52 | 53,75 | | 51,11 | 44,05 | 49,61 | 51,0 | 53,08 | 51,43 | 52,3 |
| 39 | 61,18 | 54,54 | 54,15 | 56,6 | 57,57 | 51,18 | 53,29 | 54,0 | 51,96 | 51,99 | | 55,54 | 48,07 | 54,64 | 52,4 | 50,68 | 52,26 | 51,5 |
| 40 | 57,60 | 55,38 | 55,02 | 56,0 | 57,76 | 53,52 | 56,10 | 55,8 | 57,12 | 57,96 | | 55,53 | 48,69 | 55,59 | 55,0 | 56,09 | 57,76 | 56,9 |
| 41 | 54,77 | 50,46 | 49,57 | 51,6 | 56,47 | 50,40 | 54,24 | 53,7 | 52,10 | 50,68 | | 54,93 | 46,04 | 53,44 | 51,4 | 57,70 | 49,17 | 53,4 |
| 42 | 60,56 | 55,65 | 56,62 | 57,6 | 56,84 | 50,04 | 54,11 | 53,7 | 57,72 | 55,15 | | 53,29 | 49,11 | 56,95 | 54,4 | 53,94 | 46,82 | 50,4 |
| 43 | 54,71 | 51,92 | 54,04 | 53,6 | 51,48 | 56,87 | 48,75 | 52,4 | 58,26 | 53,40 | | 57,27 | 47,47 | 56,39 | 54,6 | 53,59 | 49,49 | 51,5 |
| 44 | 64,85 | 60,43 | 52,60 | 59,3 | 59,55 | 48,04 | 56,83 | 54,8 | 59,69 | 57,08 | | 50,57 | 44,29 | 46,93 | 51,7 | 56,18 | 53,83 | 55,0 |
| 45 | 54,51 | 48,87 | 42,69 | 48,7 | 86,61 | 49,39 | 53,97 | 63,3 | 56,29 | 56,19 | | 48,44 | 38,42 | 51,10 | 50,1 | 47,04 | 45,56 | 46,3 |
| 46 | 47,51 | 49,12 | 49,99 | 48,9 | 52,93 | 56,49 | 54,87 | 54,8 | 52,63 | 52,87 | | 55,97 | 45,60 | 52,83 | 52,0 | 49,07 | 47,97 | 48,5 |
| 47 | 56,33 | 61,48 | 48,12 | 55,3 | 49,91 | 58,35 | 46,19 | 51,5 | 61,29 | 67,27 | | 69,68 | 51,44 | 61,33 | 62,2 | 49,42 | 50,50 | 50,0 |
| 48 | 44,49 | 57,84 | 51,50 | 51,3 | 49,03 | 49,02 | 55,12 | 51,1 | 54,31 | 54,64 | | 49,14 | 45,51 | 50,50 | 50,8 | 51,43 | 45,38 | 48,4 |
| 49 | 52,57 | 53,91 | 51,38 | 52,6 | 49,55 | 42,91 | 57,47 | 50,0 | 71,10 | 49,47 | | 60,87 | 54,87 | 50,96 | 57,5 | 53,76 | 55,58 | 54,7 |
| 50 | 52,32 | 53,13 | 47,54 | 51,0 | 56,20 | 53,79 | 52,86 | 54,3 | 49,15 | 53,23 | | 55,02 | 50,97 | 49,97 | 51,7 | 54,75 | 48,01 | 51,4 |
| 51 | 52,88 | 49,69 | 51,64 | 51,4 | 54,27 | 47,02 | 49,97 | 50,4 | 50,46 | 44,58 | | 46,29 | 53,62 | 46,12 | 48,2 | 47,28 | 43,05 | 45,2 |
| 52 | 50,34 | 68,28 | 57,59 | 58,7 | 57,03 | 62,40 | 59,84 | 59,8 | 73,39 | 60,70 | | 55,20 | 52,40 | 62,76 | 60,9 | 56,92 | 67,16 | 62,0 |
| 53 | 49,77 | 55,33 | 59,64 | 54,9 | 51,78 | 69,02 | 55,10 | 58,6 | 61,93 | 55,10 | | 54,73 | 50,38 | 59,28 | 56,3 | 56,27 | 51,99 | 54,1 |
| 54 | 53,35 | 50,58 | 49,04 | 51,0 | 51,75 | 50,98 | 57,28 | 53,3 | 61,86 | 58,43 | | 55,16 | 53,29 | 51,66 | 56,1 | 52,33 | 49,44 | 50,9 |
| 55 | 49,78 | 51,55 | 49,44 | 50,3 | 52,05 | 52,01 | 53,99 | 52,7 | 50,95 | 49,97 | | 52,94 | 50,77 | 52,66 | 51,5 | 50,45 | 48,94 | 49,7 |
| 56 | 53,90 | 57,34 | 56,44 | 55,9 | 50,25 | 55,38 | 52,28 | 52,6 | 49,66 | 51,92 | | 56,52 | 51,40 | 59,25 | 53,8 | 47,30 | 50,63 | 49,0 |
| 57 | 61,40 | 54,96 | 49,00 | 55,1 | 58,75 | 56,70 | 55,81 | 57,1 | 49,27 | 53,69 | | 55,41 | 47,79 | 60,29 | 53,3 | 51,05 | 47,88 | 49,5 |
| 58 | 52,12 | 53,85 | 52,13 | 52,7 | 51,17 | 50,47 | 52,90 | 51,5 | 56,77 | 50,07 | | 55,98 | 49,28 | 57,40 | 53,9 | 53,73 | 51,45 | 52,6 |
| 59 | 52,01 | 51,61 | 48,94 | 50,9 | 51,80 | 58,58 | 56,20 | 55,5 | 49,52 | 55,37 | | 53,96 | 51,06 | 54,14 | 52,8 | 55,66 | 52,82 | 54,2 |
| 60 | 57,03 | 50,85 | 46,59 | 51,5 | 56,69 | 56,04 | 54,43 | 55,7 | 48,33 | 60,24 | | 51,13 | 46,23 | 49,21 | 51,0 | 51,28 | 45,13 | 48,2 |
| 61 | 55,56 | 54,01 | 49,85 | 53,1 | 53,75 | 52,42 | 50,55 | 52,2 | 62,24 | 58,31 | | 51,53 | 49,89 | 58,38 | 56,1 | 51,58 | 56,64 | 54,1 |
| 62 | 53,30 | 53,08 | 48,04 | 51,5 | 53,71 | 49,45 | 52,62 | 51,9 | 47,76 | 53,60 | | 41,04 | 40,59 | 44,16 | 45,4 | 50,35 | 52,86 | 51,6 |
| 63 | 55,36 | 51,11 | 51,51 | 52,7 | 55,04 | 58,76 | 56,82 | 56,9 | 59,08 | 54,49 | | 50,96 | 48,10 | 55,47 | 53,6 | 48,99 | 52,59 | 50,8 |
| 64 | 56,39 | 55,41 | 57,72 | 56,5 | 64,21 | 68,81 | 71,61 | 68,2 | 64,26 | 61,81 | | 67,59 | 54,42 | 68,65 | 63,3 | 64,06 | 67,83 | 65,9 |
| 65 | 54,46 | 54,19 | 48,64 | 52,4 | 54,34 | 44,47 | 52,33 | 50,4 | 52,79 | 56,55 | | 48,01 | 50,93 | 47,16 | 51,1 | 54,82 | 45,32 | 50,1 |
| 66 | 60,25 | 56,58 | 51,72 | 56,2 | 55,76 | 44,22 | 61,78 | 53,9 | 62,75 | 62,98 | | 59,41 | 48,98 | 57,07 | 58,2 | 55,96 | 53,60 | 54,8 |
| 67 | 55,01 | 53,13 | 55,66 | 54,6 | 50,44 | 53,03 | 58,29 | 53,9 | 61,84 | 56,37 | | 52,20 | 49,67 | 58,89 | 55,8 | 54,19 | 52,99 | 53,6 |
| 68 | 58,19 | 52,54 | 51,60 | 54,1 | 55,40 | 58,13 | 55,37 | 56,3 | 53,48 | 52,25 | | 54,43 | 53,26 | 50,52 | 52,8 | 51,32 | 54,75 | 53,0 |
| 69 | 56,88 | 53,39 | 58,08 | 56,1 | 53,10 | 55,17 | 55,04 | 54,4 | 63,67 | 58,16 | | 49,24 | 48,39 | 49,71 | 53,8 | 49,45 | 47,59 | 48,5 |
| 70 | 55,46 | 53,93 | 53,03 | 54,1 | 55,60 | 54,22 | 58,06 | 56,0 | 60,83 | 60,19 | | 62,85 | 49,69 | 54,62 | 57,6 | 51,44 | 52,29 | 51,9 |
| 71 | 67,63 | 58,08 | 53,45 | 59,7 | 60,84 | 51,61 | 65,38 | 59,3 | 64,16 | 61,70 | | 49,47 | 55,50 | 52,17 | 56,6 | 55,73 | 51,10 | 53,4 |
| 72 | 56,60 | 52,89 | 54,34 | 54,6 | 55,92 | 53,90 | 52,39 | 54,1 | 56,46 | 56,83 | | 46,77 | 50,56 | 56,57 | 53,4 | 53,93 | 48,41 | 51,2 |
| 73 | 61,17 | 53,90 | 47,94 | 54,3 | 55,93 | 55,68 | 55,23 | 55,6 | 58,83 | 57,30 | | 46,96 | 51,24 | 47,60 | 52,4 | 56,20 | 51,20 | 53,7 |
| 74 | 50,18 | 55,35 | 52,26 | 52,6 | 59,29 | 45,45 | 53,15 | 52,6 | 56,64 | 49,46 | | 55,22 | 54,33 | 55,59 | 54,2 | 52,14 | 53,68 | 52,9 |
| 75 | 54,91 | 53,40 | 54,84 | 54,4 | 50,69 | 42,09 | 52,14 | 48,3 | 55,25 | 47,10 | | 59,56 | 47,82 | 64,64 | 54,9 | 46,62 | 57,94 | 52,3 |
| 76 | 51,94 | 52,62 | 47,73 | 50,8 | 45,41 | 52,71 | 51,72 | 49,9 | 57,16 | 56,82 | | 54,92 | 47,79 | 48,19 | 53,0 | 52,55 | 50,19 | 51,4 |
| 77 | 51,81 | 49,10 | 59,95 | 53,6 | 58,87 | 57,19 | 53,98 | 56,7 | 53,62 | 54,81 | | 60,74 | 54,16 | 59,33 | 56,5 | 56,56 | 57,46 | 57,0 |
| 78 | 58,16 | 58,50 | 40,44 | 52,4 | 55,63 | 48,22 | 56,10 | 53,3 | 65,63 | 51,96 | | 60,06 | 50,46 | 54,01 | 56,4 | 56,46 | 53,48 | 55,0 |
| 79 | 62,09 | 52,13 | 51,74 | 55,3 | 55,52 | 49,31 | 56,68 | 53,8 | 53,78 | 54,45 | | 55,34 | 51,31 | 48,25 | 52,6 | 51,50 | 50,90 | 51,2 |
| 80 | 60,70 | 55,84 | 52,29 | 56,3 | 54,30 | 54,04 | 53,24 | 53,9 | 58,55 | 53,85 | | 55,26 | 48,83 | 47,05 | 52,7 | 50,28 | 57,43 | 53,9 |
| 81 | 53,68 | 48,24 | 48,66 | 50,2 | 55,47 | 50,94 | 56,02 | 54,1 | 57,89 | 52,35 | | 52,71 | 47,86 | 51,58 | 52,5 | 53,31 | 53,00 | 53,2 |
| 82 | 64,42 | 56,92 | 46,44 | 55,9 | 58,24 | 49,56 | 58,04 | 55,3 | 58,07 | 63,39 | | 44,05 | 46,14 | 45,10 | 51,3 | 59,76 | 48,11 | 53,9 |
| 83 | 55,28 | 53,07 | 59,66 | 56,0 | 53,27 | 61,08 | 58,08 | 57,5 | 56,26 | 51,71 | | 65,22 | 54,44 | 59,08 | 57,3 | 51,83 | 54,29 | 53,1 |
| 84 | 54,19 | 53,33 | 46,86 | 51,5 | 54,54 | 57,06 | 48,15 | 53,3 | 53,25 | 63,16 | | 53,36 | 51,20 | 48,61 | 53,9 | 55,33 | 58,10 | 56,7 |
| 85 | 55,02 | 59,34 | 54,74 | 56,4 | 51,70 | 53,10 | 61,40 | 55,4 | 51,73 | 52,25 | | 56,14 | 51,93 | 59,64 | 54,3 | 49,12 | 52,64 | 50,9 |
| 86 | 52,37 | 51,67 | 48,08 | 50,7 | 52,32 | 56,27 | 57,61 | 55,4 | 56,24 | 55,08 | | 50,03 | 49,15 | 50,44 | 52,2 | 57,09 | 48,67 | 52,9 |
| 87 | 50,07 | 57,06 | 51,92 | 53,0 | 57,49 | 52,12 | 47,33 | 52,3 | 52,00 | 50,82 | | 55,57 | 46,93 | 57,46 | 52,6 | 53,05 | 48,30 | 50,7 |
| 88 | 59,09 | 54,16 | 45,21 | 52,8 | 51,62 | 50,17 | 51,84 | 51,2 | 52,91 | 53,97 | | 48,50 | 50,17 | 43,87 | 49,9 | 49,24 | 48,73 | 49,0 |
| Mean | 54,7 | 53,8 | 51,6 | 53,4 | 54,3 | 52,5 | 55,0 | 53,9 | 56,3 | 54,5 | | 53,9 | 49,2 | 53,8 | 53,5 | 53,1 | 51,7 | 52,4 |
| SD | 4,4 | 3,4 | 3,9 | 2,5 | 5,0 | 4,8 | 4,1 | 3,1 | 4,8 | 4,2 | | 4,8 | 3,4 | 4,8 | 2,9 | 3,9 | 4,7 | 3,6 |
